# Small-Pox Statistics

**Published:** 1873-01

**Authors:** 


TABULAR STATEMENT of 22866 Vaccinations and Re-Vaccinations at different periods of life, in which the results were carefully observed and recorded by Physicians of the Relief and Aid Society, during the winter of 1871–2. The number of cases of Small-Pox occurring after successful and unsuccessful Vaccinations are also shown.

### SUCCESSFUL VACCINATIONS.

| NATIONALITY. | Under 5 yrs. | | | Small-Pox. | | | 5 to 10 yrs. | | | Small-Pox. | | | 10 to 20 yrs. | | | Small-Pox. | | | 20 yrs. & upward. | | | Small-Pox. | | |
|---|---|---|---|---|---|---|---|---|---|---|---|---|---|---|---|---|---|---|---|---|---|---|---|---|
| | 1st | 2d | 3d | 1st | 2d | 3d | 1st | 2d | 3d | 1st | 2d | 3d | 1st | 2d | 3d | 1st | 2d | 3d | 1st | 2d | 3d | 1st | 2d | 3d |
| American | 250 | 59 | 6 | 3 | 2 | .... | 61 | 114 | 6 | 2 | 1 | .... | 50 | 167 | 34 | 3 | 6 | 1 | 53 | 236 | 102 | 7 | 5 | 2 |
| German | 399 | 148 | 18 | 3 | .... | 2 | 95 | 353 | 26 | 6 | 3 | 1 | 51 | 434 | 82 | 2 | 6 | 1 | 77 | 1144 | 304 | 18 | 45 | 21 |
| Irish | 1007 | 282 | 47 | 7 | 1 | 4 | 233 | 691 | 119 | 2 | 4 | 4 | 131 | 840 | 266 | 7 | 17 | 12 | 239 | 1381 | 322 | 45 | 147 | 38 |
| German-American | 386 | 191 | 13 | 4 | .... | .... | 63 | 366 | 29 | 2 | 16 | 1 | 40 | 377 | 41 | 10 | 30 | 2 | 3 | 26 | 5 | 1 | 1 | 1 |
| Irish-American | 225 | 153 | 2 | 1 | 2 | .... | 42 | 306 | 22 | 1 | 9 | .... | 14 | 395 | 66 | 5 | 26 | 4 | 5 | 39 | 11 | 4 | 9 | 2 |
| Colored-American | 54 | 4 | 2 | .... | .... | .... | 26 | 15 | .... | .... | .... | .... | 8 | 23 | 3 | .... | 1 | .... | 24 | 55 | 9 | 6 | .... | 1 |
| Swedish | 158 | 31 | 5 | 3 | .... | .... | 67 | 79 | 12 | 1 | 2 | .... | 31 | 106 | 20 | 4 | 2 | 1 | 55 | 202 | 87 | 9 | 11 | 2 |
| Norwegian | 139 | 19 | 3 | .... | .... | .... | 54 | 80 | 10 | 1 | .... | .... | 33 | 90 | 21 | 1 | .... | .... | 41 | 132 | 82 | 2 | 7 | 4 |
| English | 98 | 20 | 1 | 1 | .... | .... | 28 | 53 | 1 | .... | .... | .... | 12 | 64 | 17 | .... | 3 | 1 | 25 | 87 | 30 | 9 | 13 | 2 |
| French | 41 | 9 | .... | 4 | .... | .... | 8 | 24 | 2 | .... | .... | .... | 1 | 22 | 7 | .... | 2 | .... | 8 | 26 | 8 | 2 | 1 | .... |
| Scotch | 25 | 12 | .... | .... | .... | .... | 15 | 18 | 3 | .... | .... | .... | 5 | 23 | 6 | .... | 1 | .... | 6 | 32 | 7 | 2 | 2 | .... |
| Scandinavian | 31 | 29 | .... | .... | .... | .... | .... | 25 | 5 | .... | .... | .... | 1 | 31 | 2 | .... | .... | .... | .... | 55 | 6 | .... | 1 | .... |
| Italian | 38 | 10 | .... | .... | .... | .... | 6 | 24 | .... | .... | .... | .... | 3 | 36 | 3 | .... | .... | .... | 7 | 42 | 6 | 2 | 3 | 1 |
| Canadian | 16 | 9 | 2 | .... | 1 | .... | 6 | 23 | 3 | .... | .... | .... | .... | 29 | 9 | .... | .... | .... | 1 | 35 | 9 | .... | 3 | .... |
| Bohemian | 22 | 7 | .... | .... | .... | .... | 6 | 31 | .... | .... | .... | .... | .... | 17 | 1 | .... | .... | .... | 4 | 50 | 2 | .... | .... | .... |
| Prussian | 7 | 1 | .... | .... | .... | .... | 2 | 2 | 3 | .... | .... | .... | 1 | 7 | 1 | .... | .... | .... | 5 | 5 | .... | .... | 2 | .... |
| Danish | 11 | .... | .... | .... | .... | .... | 4 | 3 | 1 | .... | .... | .... | 4 | 3 | .... | .... | .... | .... | 8 | 3 | 5 | .... | .... | .... |
| Belgian | 1 | .... | .... | .... | .... | .... | .... | .... | .... | .... | .... | .... | .... | .... | .... | .... | .... | .... | .... | .... | 1 | .... | .... | .... |
| Holland | 5 | 3 | 1 | .... | .... | .... | 1 | 7 | .... | .... | .... | .... | .... | 4 | 1 | .... | .... | .... | .... | 8 | 2 | .... | .... | .... |
| Denmark | 2 | 3 | .... | .... | .... | .... | .... | 2 | .... | .... | .... | .... | .... | 1 | .... | .... | .... | .... | .... | 27 | 1 | .... | 1 | .... |
| Russian | 2 | 2 | 12 | .... | .... | .... | 12 | .... | 8 | .... | .... | .... | 8 | 4 | 4 | .... | .... | .... | 24 | 4 | 22 | .... | .... | .... |
| Jews | 7 | 1 | .... | .... | .... | .... | 1 | 3 | .... | .... | .... | .... | 1 | 4 | 1 | .... | .... | .... | 1 | 8 | .... | .... | .... | .... |
| Miscellaneous | 24 | 6 | 5 | .... | .... | .... | 2 | 17 | .... | .... | .... | .... | 3 | 20 | 1 | .... | .... | .... | 6 | 27 | 3 | .... | 4 | .... |
| Totals | 2948 | 999 | 117 | 26 | 6 | 6 | 732 | 2236 | 250 | 15 | 37 | 6 | 397 | 2697 | 586 | 32 | 94 | 22 | 592 | 3624 | 1024 | 107 | 355 | 74 |

Total successful Vaccination and Re-Vaccination, . . . . . . . . . . . . . . 16202
Total Small-Pox, after successful Vaccination and Re-Vaccination, . . . . . . . . . . . 780

In addition to the above, we have records of 4197 cases which have been excluded, on the ground of incompleteness, from the general statement, and which, if added, would show a total of 27063 cases.

Among the Swedish, in one case, there were three good scars on each arm; still the patient had Small-Pox very bad. (Dr. Strong's Collection.)
Among the English, two cases had Small-Pox, and were afterward successfully Vaccinated. (Dr. Clark's Collection.)
Among the Norwegian, five cases of Small-Pox after successful Vaccination, and one case fatal. (Dr. Clark's Collection.)
Among the Irish, seven cases successfully Vaccinated after they had had Small-Pox, and twelve cases had Small-Pox after successful Vaccination.
Among the American, one case successfully Vaccinated after it had had Small-Pox. (Dr. Clark's Collection.)
Among the Irish, one case of Small-Pox after successful Vaccination, and twenty-one before Vaccination. (H. J. Burlingame.)
Among the German, one case of Small-Pox after 2d unsuccessful Vac., and five before Vac., and three cases after successful Vac. (H. J. Burlingame.)
Among the Swedish, six cases of Small-Pox before Vaccination. (H. J. Burlingame.)
Among the Norwegian, two cases of Small-Pox before successful Vaccination. (H. J. Burlingame.)

### UNSUCCESSFUL VACCINATIONS.

| NATIONALITY. | Under 5 yrs. | | | Small-Pox. | | | 5 to 10 yrs. | | | Small-Pox. | | | 10 to 20 yrs. | | | Small-Pox. | | | 20 yrs. & upward. | | | Small-Pox. | | |
|---|---|---|---|---|---|---|---|---|---|---|---|---|---|---|---|---|---|---|---|---|---|---|---|---|
| | 1st | 2d | 3d | 1st | 2d | 3d | 1st | 2d | 3d | 1st | 2d | 3d | 1st | 2d | 3d | 1st | 2d | 3d | 1st | 2d | 3d | 1st | 2d | 3d |
| American | 22 | 37 | 1 | 4 | 4 | .... | 10 | 56 | 8 | 4 | .... | .... | 11 | 59 | 28 | 5 | .... | 2 | 13 | 118 | 90 | 11 | 11 | 16 |
| German | 40 | 96 | 10 | .... | .... | 1 | 18 | 152 | 33 | 6 | 2 | 3 | 5 | 212 | 63 | .... | 8 | 2 | 30 | 844 | 256 | 18 | 82 | 16 |
| Irish | 57 | 145 | 20 | 1 | 4 | 2 | 35 | 286 | 56 | 11 | 3 | 2 | 26 | 316 | 95 | 15 | 9 | .... | 67 | 749 | 198 | 53 | 127 | 16 |
| German-American | 53 | 75 | 2 | 2 | 2 | .... | 18 | 197 | 17 | 5 | 18 | .... | 15 | 285 | 33 | 6 | 35 | 1 | 1 | 37 | 9 | 1 | 6 | 2 |
| Irish-American | 21 | 41 | 3 | 2 | 1 | .... | 3 | 71 | 10 | ... | 9 | .... | 4 | 151 | 23 | 3 | 15 | 1 | .... | 15 | 7 | .... | 4 | .... |
| Colored-American | 7 | 18 | .... | 4 | 6 | .... | 2 | 13 | 2 | 1 | .... | 1 | .... | 20 | 11 | .... | .... | .... | 14 | 40 | 24 | 12 | 4 | .... |
| Swedish | 9 | 26 | 2 | .... | .... | .... | 1 | 39 | 6 | .... | 1 | 1 | 5 | 47 | 20 | 4 | 5 | ... | 13 | 103 | 31 | 9 | 11 | 1 |
| Norwegian | 11 | 8 | 1 | .... | .... | .... | 4 | 13 | 2 | .... | .... | .... | 2 | 14 | 9 | 1 | .... | .... | 1 | 43 | 22 | .... | 2 | .... |
| English | 7 | 5 | .... | .... | .... | .... | 1 | 17 | 2 | .... | 1 | .... | 2 | 28 | 6 | 1 | .... | .... | 5 | 29 | 25 | 1 | 8 | 1 |
| French | .... | .... | 1 | .... | .... | .... | .... | 1 | .... | .... | .... | .... | .... | 8 | 1 | .... | .... | .... | 1 | 18 | 9 | .... | 3 | 1 |
| Scotch | 4 | 6 | .... | .... | .... | .... | 1 | 9 | 1 | 1 | .... | .... | .... | 5 | 3 | .... | 1 | .... | 5 | 25 | 12 | 4 | 4 | 1 |
| Scandinavian | 3 | 6 | 1 | .... | .... | .... | 1 | 9 | 2 | .... | 1 | .... | .... | 37 | 3 | .... | .... | 3 | .... | 78 | 8 | .... | 6 | .... |
| Italian | 2 | 9 | .... | .... | .... | .... | .... | 8 | .... | .... | .... | .... | 2 | 15 | 4 | .... | 2 | .... | 3 | 22 | 9 | 1 | 4 | 2 |
| Canadian | 3 | 2 | .... | 1 | 1 | .... | .... | 3 | 2 | .... | .... | .... | .... | .... | 1 | .... | .... | .... | .... | 6 | 5 | .... | 1 | .... |
| Bohemian | 3 | 11 | .... | .... | .... | .... | 1 | 9 | 1 | .... | .... | .... | .... | 9 | 7 | .... | .... | .... | .... | 28 | 3 | .... | .... | .... |
| Prussian | .... | .... | .... | .... | .... | .... | .... | .... | .... | .... | .... | .... | .... | .... | .... | .... | .... | .... | .... | 1 | 1 | .... | .... | .... |
| Danish | .... | .... | 1 | .... | .... | .... | .... | .... | .... | .... | .... | .... | .... | .... | .... | .... | .... | .... | 2 | .... | 1 | .... | .... | .... |
| Belgian | .... | 1 | .... | .... | .... | .... | .... | .... | .... | .... | .... | .... | .... | .... | .... | .... | .... | .... | .... | .... | 1 | .... | .... | .... |
| Holland | .... | .... | .... | .... | .... | .... | .... | 3 | 2 | .... | .... | .... | .... | 1 | 1 | .... | .... | .... | .... | 3 | .... | .... | .... | .... |
| Denmark | .... | 3 | .... | .... | .... | .... | .... | .... | .... | .... | .... | .... | .... | .... | 1 | .... | .... | .... | .... | 2 | .... | .... | .... | .... |
| Russian | 10 | 8 | .... | 1 | .... | 2 | 2 | 12 | 6 | .... | 4 | 2 | 4 | 10 | 12 | .... | 2 | 4 | 2 | 20 | 4 | .... | 6 | 2 |
| Jews | .... | 2 | .... | .... | .... | .... | .... | 1 | .... | .... | .... | .... | .... | .... | 1 | .... | .... | .... | 2 | 3 | .... | 1 | .... | .... |
| Miscellaneous | 1 | 4 | 5 | .... | .... | ... | 1 | 8 | .... | .... | .... | .... | .... | 9 | 2 | .... | 1 | .... | 1 | 17 | 4 | 1 | 1 | .... |
| Totals | 253 | 503 | 47 | 15 | 18 | 5 | 98 | 907 | 150 | 28 | 39 | 9 | 76 | 1226 | 324 | 35 | 78 | 13 | 160 | 2201 | 719 | 112 | 280 | 58 |

Total unsuccessful Vaccination and Re-Vaccination, . . . . . . . . . . . . . . 6664
Total Small Pox after unsuccessful Vaccination and Re-Vaccination, . . . . . . . . . . . . 690

Among the Americans, one adult had been several times unsuccessfully Vaccinated, and had Small-Pox. Another case under 5 years was successfully Vaccinated, and at same time had Small-Pox. (Dr. Chas. White's Collection.)

Among the Scandinavians, two successful Vaccinations and Small-Pox occurred in one individual. (Dr. Chas. White's Collection.)

Among the English, one case badly pitted, and second Vaccination successful. (Dr. Chas. White's Collection.)

Among the German-American, one case Small-Pox at 7 years, but a successful Vaccination occurred afterward between 10 and 20 years. (Dr. Chas. White's Collection.)

One case had Small-Pox twice. (Dr. Leonard's Collection.)

Total number of cases of severe Small-Pox, 55. General average time occurring after a successful Vaccination—1st Vaccination, 7 years; 2d Vaccination, 11 5-6 years; 3d Vaccination, 15 3-4 years. (Dr. Bockin's Collection.)

